# Toward Electrically Tunable, Lithography-Free, Ultra-Thin Color Filters Covering the Whole Visible Spectrum

**DOI:** 10.1038/s41598-018-29544-x

**Published:** 2018-07-27

**Authors:** Majid Aalizadeh, Andriy E. Serebryannikov, Amin Khavasi, Guy A. E. Vandenbosch, Ekmel Ozbay

**Affiliations:** 1 Department of Electrical and Electronics Engineering, Bilkent University, Ankara, 06800 Turkey; 2 Nanotechnology Research Center (NANOTAM), Bilkent University, Ankara, 06800 Turkey; 3 ESAT-TELEMIC, Katholieke Universiteit Leuven, 3000 Leuven, Belgium; 4 Faculty of Physics, Adam Mickiewicz University, 61-614 Poznan, Poland; 5 Electrical Engineering Department, Sharif University of Technology, Tehran, 11155-4363 Iran; 6 National Nanotechnology Research Center (UNAM), Bilkent University, Ankara, 06800 Turkey; 7 Department of Physics, Bilkent University, Ankara, 06800 Turkey

## Abstract

The possibility of real-time tuning of optical devices has attracted a lot of interest over the last decade. At the same time, coming up with simple lithography-free structures has always been a challenge in the design of large-area compatible devices. In this work, we present the concept and the sample design of an electrically tunable, lithography-free, ultra-thin transmission-mode color filter, the spectrum of which continuously covers the whole visible region. A simple Metal-Insulator-Metal (MIM) cavity configuration is used. It is shown that using the electro-optic dielectric material of 4-dimethyl-amino-N-methyl-4-stilbazoliumtosylate (DAST) as the dielectric layer in this configuration enables efficient electrical tuning of the color filter. The total thickness of the structure is 120 nm, so it is ultra-thin. The output color gets tuned from violet to red by sweeping the applied voltage from −12 to +12 Volts (V). We present an in-detail optimization procedure along with a simple calculation method for the resonance wavelength of the MIM cavity that is based on circuit theory. Such power-efficient structures have a large variety of potential applications ranging from optical communication and switching to displays and color-tunable windows.

## Introduction

The electromagnetic (EM) response of an optical device depends on the dimensions and material parameters of its components. This often imposes a limitation on their tuning functionality in a way that the EM response gets determined at the stage of fabrication, since new dimensions and/or materials are required to obtain a new EM response. To obtain the possibility of real-time tuning of the EM response of optical structures after the fabrication, dynamically tunable scenarios are required to be implemented. Among different ways of tuning, tuning with variable voltage (electrical tuning) has always been the most desirable and has been the topic of extensive research over the last decade. For this purpose, different methods and materials have been suggested. Utilizing electro-optically active materials is one of the most common methods for electrical tuning of optical structures. Optical behavior of these materials is modified by applying voltage. Liquid crystals^1–4^, organic crystals^5–7^, graphene^8–11^, heavily doped compounds like Al-doped ZnO (AZO)^12^ or n-type Indium Antimonide (n-InSb)^13^, and transparent conductive oxides like Indium Tin Oxide (ITO)^14–19^ or Indium Zinc Oxide (IZO)^20^ are common materials used for this purpose. The choice of an electro-optic (EO) material depends on the design and the operating wavelength range of the optical structure. It is noteworthy that micro-electro-mechanical systems (MEMS) or nano-electro-mechanical systems (NEMS) have also been used to provide optical devices with the possibility of real-time controlling^21,22^.

Recently, several works have been reported, in which ITO is used to electrically tune the reflection spectrum of the optical structures, in particular, the one of the reflect-array metasurfaces^14–19^. Some works have been dedicated to the use of ITO as the tunable material to actively steer the beam reflected from metasurfaces^18,19^. The structures involving ITO as the active material generally use it in the configuration of Metal-Oxide-Semiconductor (MOS) capacitor, so that by applying voltage, the carrier concentration of ITO changes in the vicinity of the ITO-oxide interface. These changes lead to those in the plasma frequency of the Drude model of the permittivity of ITO, and thus modulate the permittivity and the refractive index profile of ITO^23^. This is how the optical response of such structures gets tuned by the application of voltage.

A number of works have also used graphene as the active material^8–11^. The tuning in these works has been possible due to the shift in the Fermi energy level of graphene, which is obtainable by applying a bias voltage, so that the optical response of graphene and the whole structure is changed.

Color generators represent a wide class of the optical devices having various applications including displays, printing technologies, imaging, and light emitters. Some structural color generators are available in nature in specific types of insects, birds, and animals, such as butterfly wings^24–28^. Initial artificial color displays have employed liquid crystals, and produce the three main colors, red, green, and blue^29–31^. Recently, metamaterials have been introduced as a new platform that enables the realization of color displays with higher compactness, better resolution, better power-efficiency, and more flexibility in design-based color tunability. There is a lot of works demonstrating color filters without the possibility of real-time tuning^32–39^. At the same time, tunable color filters working in the transmission or reflection mode have also been reported; some of which are dynamically controllable by the polarization of incident light^40–42^, while some others are electrically tunable^1,4,13,43^. For example, it has been suggested to use a polarization-tailored dichroic resonator combined with a twisted nematic liquid crystal^44^. However, in this case, lithography is required in the fabrication process.

In most of the earlier works, lithography is typically required as a part of fabrication process, which is the main barrier to mass production. It brings many limitations such as significantly restricting the area of samples, decreasing the throughput, and increasing the cost of fabrication. Therefore, coming up with a lithography-free design is of great importance for the purpose of large-area production and cost reduction. A lithography-free approach has been recently proposed in a few works for color filters, which cannot be tuned in real time^18,19^. These series of lithography-free bandpass filters are based on the MIM cavities and work in the transmission mode.

In this work, we propose an electrically tunable, lithography-free, ultra-thin color filter. It is designed by utilizing the EO dielectric organic crystal of 4-dimethyl-amino-N-methyl-4-stilbazoliumtosylate (DAST) as the insulator layer and Silver (Ag) as the metal layer, in the simple MIM cavity^45^ configuration. The total thickness of the structure is only 120 nm, where the thickness of layers (metal, insulator, and metal) is 25, 70, and 25 nm, from the top to the bottom. Description of optimization procedure along with conceptual analysis is presented. Moreover, a simple approach has been suggested for the determination of the resonance wavelength by the use of circuit theory, which has not yet been applied to the MIM structures, to the best of our knowledge. The passband region of the designed filter continuously covers the whole visible region by tuning the applied voltage, so that the output color changes from violet to red by changing the voltage from −12 to +12 volts. The device has the capability of functioning with negative voltages, which makes it relatively power-efficient. It can have applications in optical communication and switching, dynamic displays, holograms, imaging devices, light emitting structures, and smart color-tunable windows.

It has to be mentioned that in contrast with ref.^46^, in which MIM waveguides with the localized input and output were suggested as color filters, in our work operation in the transmission mode allows us using incident beams of rather arbitrary width. Moreover, our color filter enables continuous tuning over the whole visible range owing to a linear change of refractive index of the used EO material, compared to^46^.

## Calculation and Analysis

**Figure 1:**
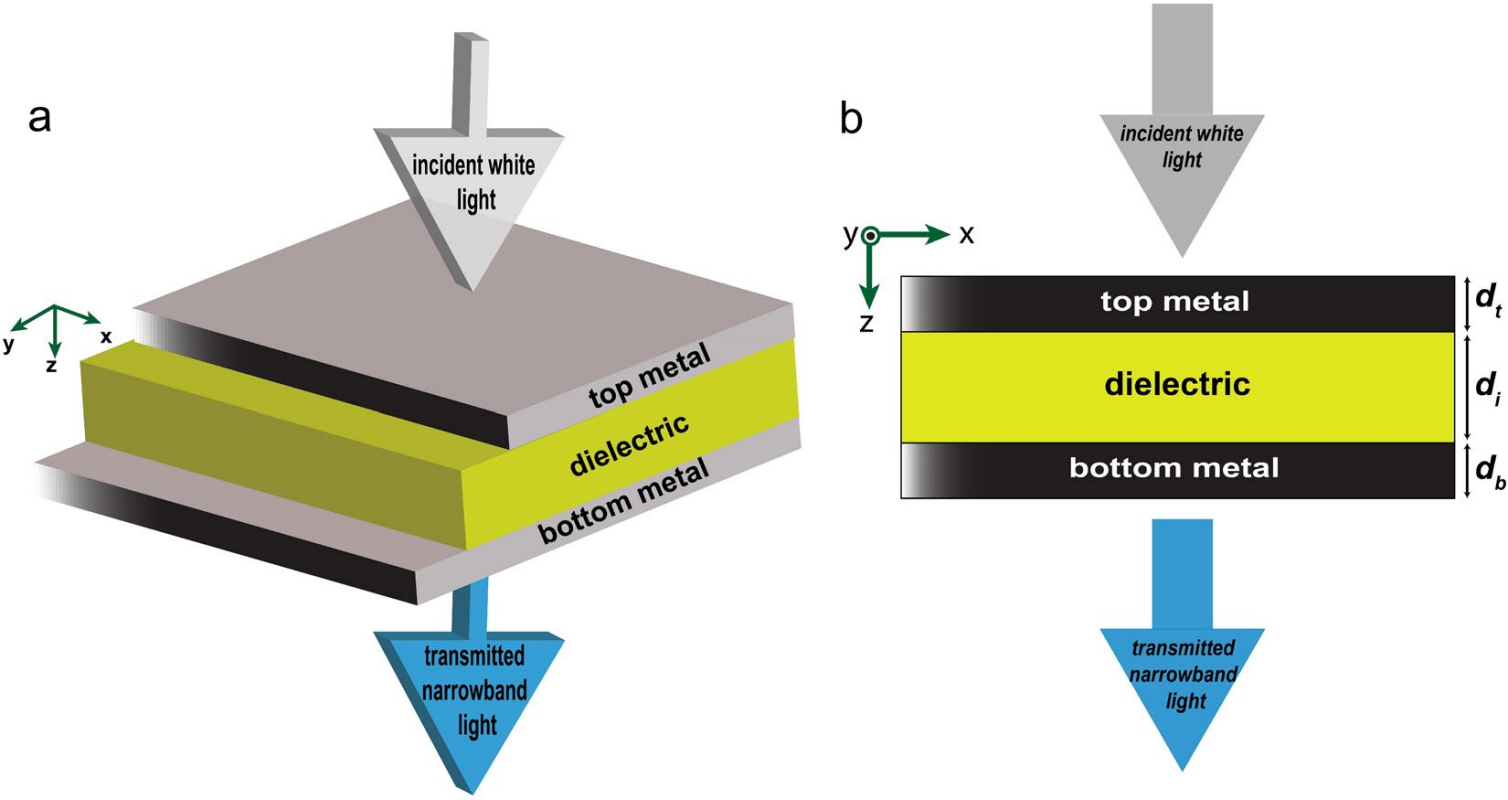
(**a**) 3D schematic of the MIM filter and, (**b**) the cross-section of the structure in (x,z)-plane. The names assigned to the thickness of layers are demonstrated in (**b**).

Figure 1(a) depicts the 3D schematic of the structure. It consists of two metal layers and an insulator layer sandwiched between them. Both metal layers should be thin so that the top metal layer allows the incident light couple into the structure, and the bottom layer allows the light couple out of the structure and have a significant transmission. In other words, the MIM configuration acts like a Fabry-Perot cavity, in which the metal layers play the role of partially transparent mirrors and the dielectric layer fills the space between the mirrors. The physical phenomenon taking place in this cavity is that the incident light is partially reflected from the top metal-air interface, and partially penetrates into metal, and eventually penetrates into the cavity. The light is reflected back and forth between two semi-transparent metals and travels in the dielectric region of the MIM resonator. In each reflection, some part of light transmits through the bottom metal layer, eventually leading a significant portion of the incident light to be transmitted through the structure. Because of the Fabry-Perot-like behavior of the MIM cavity, not all the spectrum of the incident light gets trapped inside the cavity. Only spectral components corresponding to the resonance wavelength of the MIM cavity get trapped and eventually transmitted. The resonance wavelength of the MIM cavity, which determines the narrow transmission spectrum of this MIM filter, follows the equation^47^:

1 $$2(\frac{2\pi }{{\lambda }_{res}}){n}_{i}{d}_{i}+{\varphi }_{b}+{\varphi }_{t}=2\pi m,$$

where *λ*_*res*_, *n*_*i*_, and *d*_*i*_ denote the resonance wavelength of the MIM filter, the refractive index, and the thickness of the dielectric layer, respectively. The second and third terms in the left-hand side, *ɸ*_*b*_ and *ɸ*_*t*_, denote the phase shift due to reflection from the bottom and the top metal layers, respectively. Finally, *m* in the right-hand side is an integer that determines the order of the MIM cavity mode. For the light to be trapped inside the MIM cavity, the phase condition in equation (1) must be satisfied. This means that the phase accumulated in a full round-trip inside the cavity, which includes the effect of reflection from the metal layers (left-hand side of equation (1)) should be an integer multiple of 2*π* to enable the constructive interference of partially transmitted waves in each full round trip. Eventually, this constructive interference leads to the enhanced transmission of light through the cavity.

To calculate the transmission for the studied structures, the Transfer Matrix Method (TMM) is adopted in this work. The TMM calculations are verified by using Finite Difference Time Domain (FDTD) simulations performed by Lumerical FDTD solutions, a commercial software package^48^. Details of the TMM calculations are presented in the Supplementary Information.

Now, we present the circuit model of the studied structure, which is built by using the transmission line theory. It is noteworthy that circuit models are quite universal. They have successfully been used in analysis and design of various optical^49^, terahertz^9,10,50^, and microwave^51^ structures. In our case, the circuit model significantly simplifies the calculation of the reflection phases for the bottom and the top metal layers, i.e., *ɸ*_*b*_ and *ɸ*_*t*_. The rigorous electromagnetic approach is significantly more complex compared to the circuit theory approach we use here. It has to be mentioned that, to our knowledge, the circuit theory approach has not been applied in earlier works for the reflection phase calculations in MIM structures. In addition to the simplicity, it allows us to predict the resonance wavelength with perfect accuracy, as will be demonstrated in the next section.

**Figure 2:**
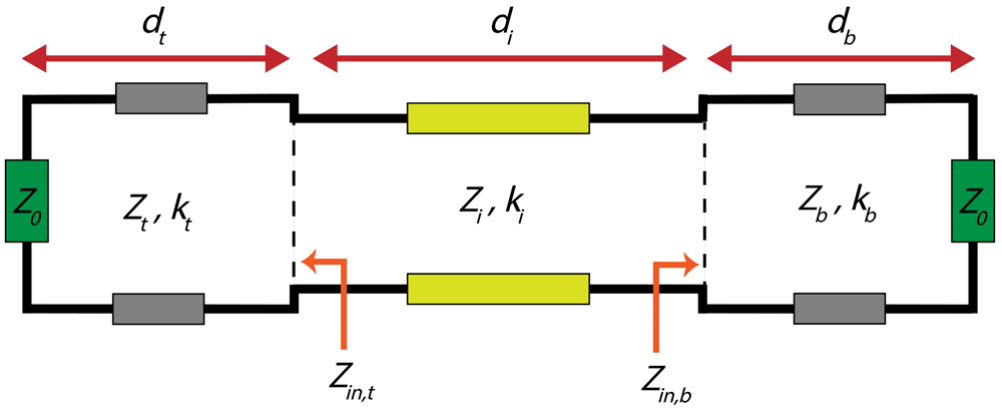
Circuit model of the structure using the transmission line theory. The incident light approaches from the left side.

The circuit model of the proposed structure is presented in Fig. 2. *Z*_*t*_, *k*_*t*_, and *d*_*t*_ are the normalized (to free space impedance) characteristic impedance, propagation constant, and the thickness of the transmission line for the top metal layer. By changing the subscripts of the same parameters from *t* to *i* or *b*, the similar parameters are introduced for the dielectric or the bottom metal layer, respectively. Z_0_ is the free-space impedance and its normalized value is 1.

In order to calculate the reflection coefficient of the top and bottom metal layers, one needs the input impedances seen from the metal-dielectric interfaces towards the metal layers. The input impedance seen from the interface of the top metal layer and the dielectric layer is denoted by *Z*_*in*,*t*_ in the Fig. 2. *Z*_*in*,*b*_ in this figure is the same parameter, but corresponding to the bottom metal layer. *Z*_*in*,*t*_ is indeed the transformed impedance of air through the top metal layer. It is obtained using the following equation^52^:

2 $${Z}_{in,t}={Z}_{t}\frac{1-j{Z}_{t}\,\tan ({k}_{t}{d}_{t})}{{Z}_{t}-j\,\tan ({k}_{t}{d}_{t})}\cdot $$

*Z*_*in*,*b*_ can be calculated similarly, just by changing the subscripts from *t* to *b* in equation (2).

The reflection coefficients of the top metal layer and the bottom metal layer are named as *r*_*t*_ and *r*_*b*_, respectively. *r*_*t*_ can be calculated as follows:

3 $${r}_{t}=\frac{{Z}_{in,t}-{Z}_{i}}{{Z}_{in,t}+{Z}_{i}}\cdot $$

*r*_*b*_ is calculated similarly, by replacing *Z*_*in*,*t*_ with *Z*_*in*,*b*_. *r*_*t*_ and *r*_*b*_ are, in general case, complex numbers which can be written in the phasor form as follows:

4a $${r}_{t}=|{r}_{t}|\,\angle \,{\varphi }_{t},$$

4b $${r}_{b}=|{r}_{b}|\,\angle \,{\varphi }_{b}.$$

**Figure 3:**
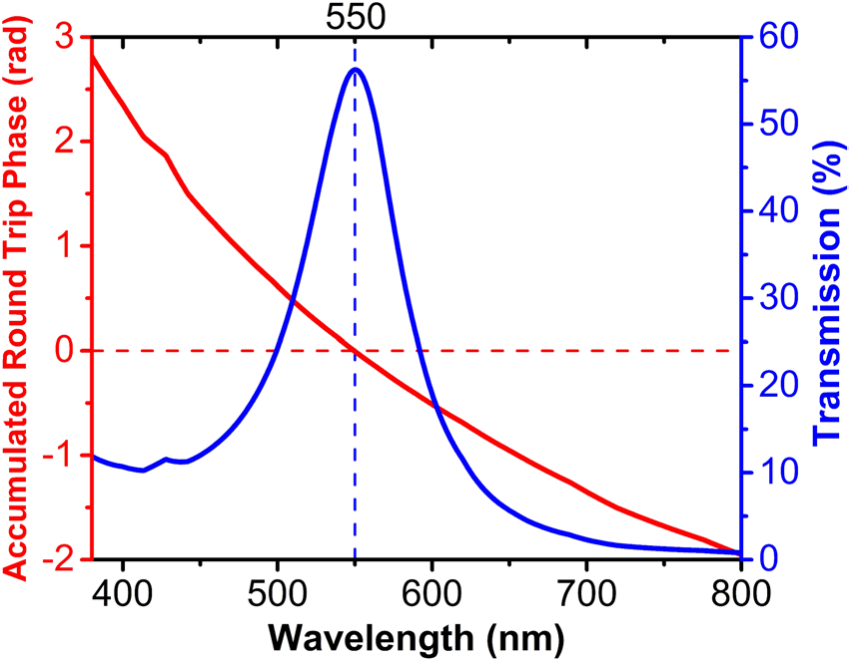
The transmission spectrum (blue line) of the MIM structure with the metal layers chosen as Ag and the values of *n*_*i*_, *d*_*t*_, *d*_*i*_ and *d*_*b*_ being 2.2, 25 nm, 70 nm, and 25 nm, respectively; the corresponding vertical axis being on the right side of the plot in blue color. The accumulated round-trip phase (red line) according to equation (1) calculated by using the proposed circuit model; the corresponding vertical axis being on the left side of the plot in red color.

Now, the calculation methods are elevated in detail, so we can calculate the transmission spectrum of an MIM filter. The top and the bottom metal layers both are chosen to be Ag with the thickness of 25 nm. The refractive index and the thickness of the dielectric layer are 2.2 and 70 nm, respectively. The thickness of the dielectric layer is chosen to have the resonance wavelength and the transmission band at the visible range. Figure 3 shows the transmission spectrum of this MIM structure, which is calculated by using the TMM method.

To show that the resonance wavelength of the MIM cavity-based filter is correctly determined by (1), the total phase accumulated in a round-trip inside the cavity, *ɸ*_*tot*_, which is given by the sum of the three terms in the left-hand side of (1), is calculated using the circuit model, as explained above. The obtained *ɸ*_*tot*_ of this structure is shown in Fig. 3 in red color together with the transmission spectrum shown in blue color. As follows from (1), *ɸ*_*tot*_ must be equal to an integer multiple of 2*π*, *m* ≥ 0. In Fig. 3, we have shown the point, at which this condition is satisfied for *m* = 0. As seen from Fig. 3, the resonance wavelength found from the phase condition perfectly matches the resonance wavelength (or the peak wavelength) of the transmission spectrum and is equal here to 550 nm.

**Figure 4:**
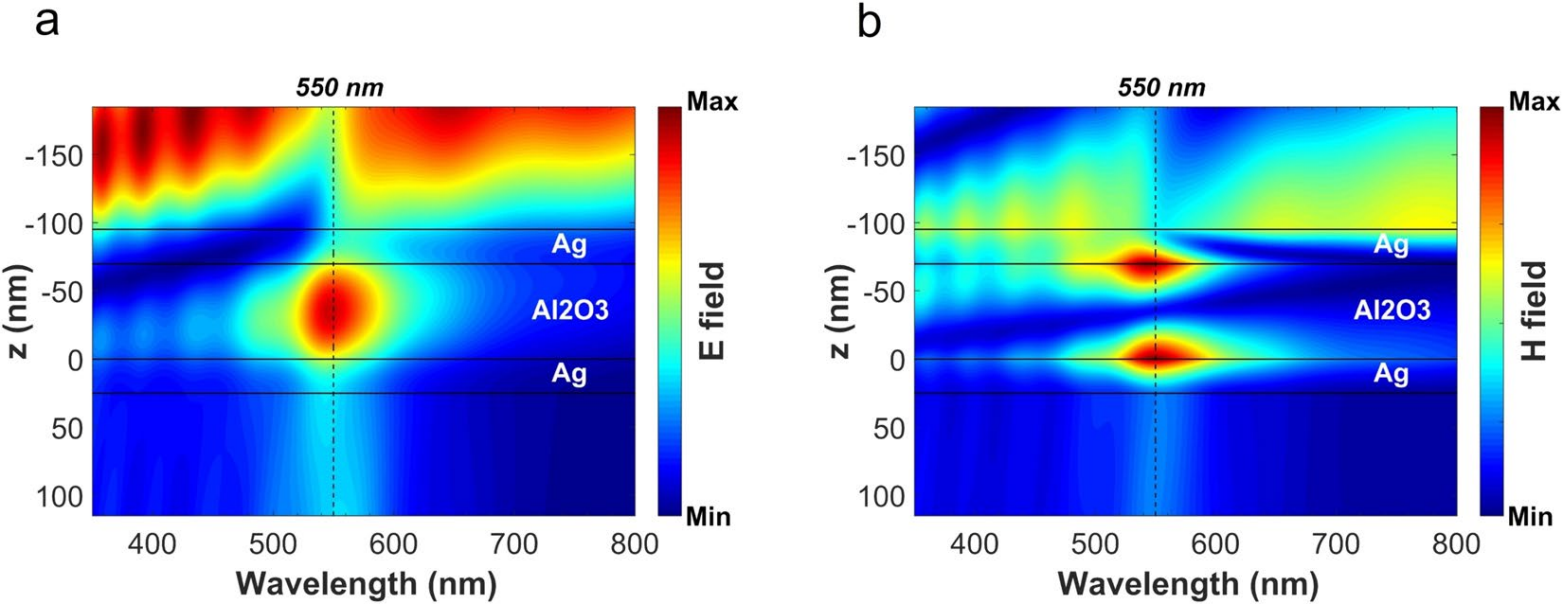
Contour plots of the magnitude of (**a**) the electric and, (**b**) the magnetic field in the studied structure, the transmission spectrum of which is shown in Fig. 3, versus the wavelength. The vertical dashed line crosses the wavelength of 550 nm, which corresponds to the resonance of this MIM filter.

For a better understanding of the electromagnetic phenomena occurring inside the structure, the magnitude of the electric (E) and magnetic (H) field distributions along the z-axis (normal to the layers of the structure), versus wavelength are shown in Fig. 4(a,b), respectively. The dashed vertical lines in these figures correspond to the resonance wavelength which is equal to 550 nm. From both figures, it is clearly seen that the structure supports a cavity resonance mode at this wavelength, which is trapped inside the dielectric layer. It becomes more obvious while looking at the E field distribution.

The other explanation of this phenomenon is that the field distributions convey the existence of the even mode supported by the MIM resonator at the resonance wavelength (see Fig. 4(b)). However, since the metal layers are not infinite in terms of their thickness, this supported mode is a leaky mode which can be coupled to the incident and transmitted plane waves, leading to enhanced transmission at the resonance wavelength.

## Optimization of the structure

In this section, we will investigate the effect of the dimensions and materials on the transmission spectrum and optimize the proposed structure.

**Figure 5:**
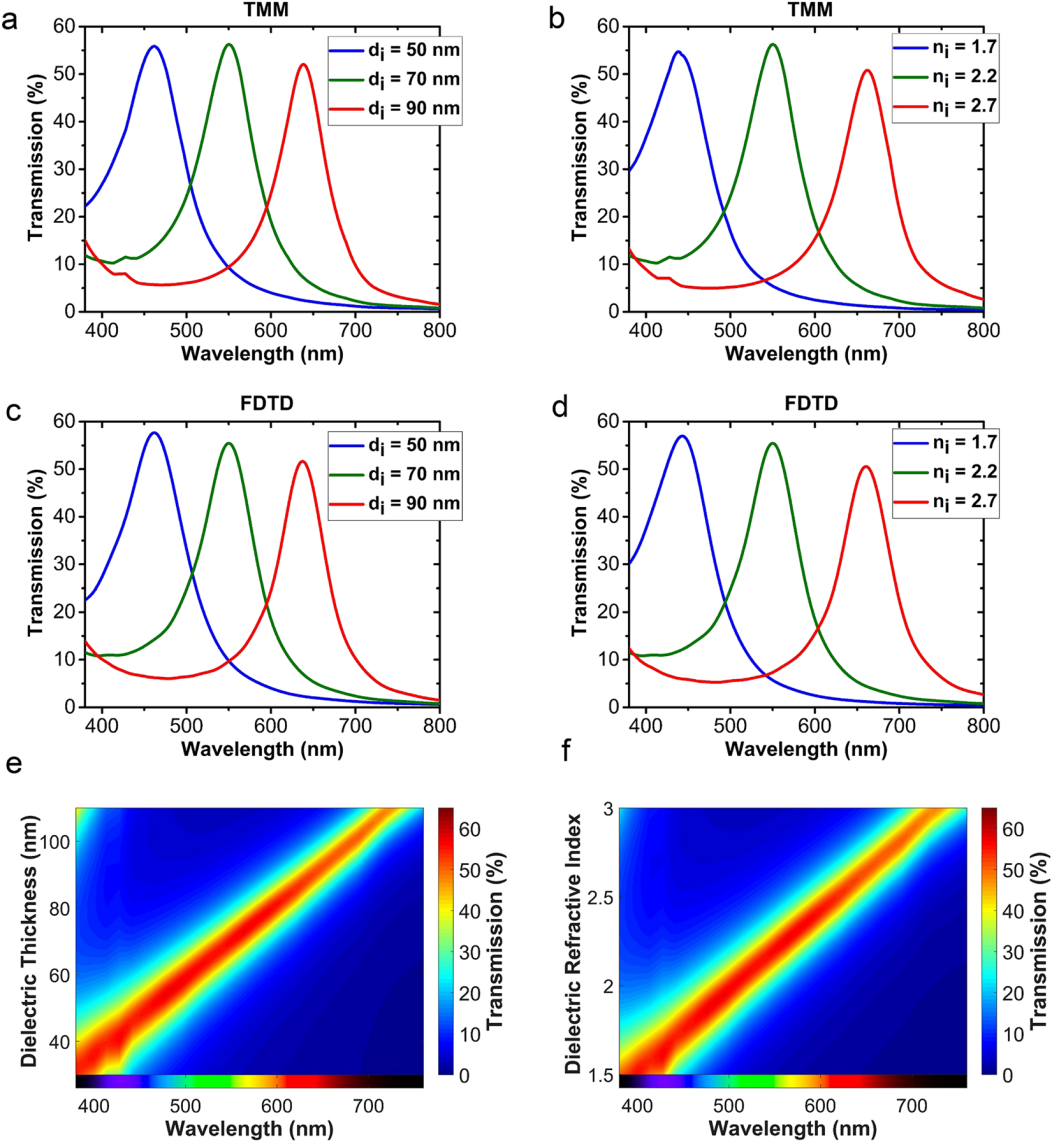
The transmission spectrum of the MIM filter calculated for different values of *d*_*i*_ when *n*_*i*_ = 2.2 by using (**a**) the TMM, and, (**c**) the FDTD method, and for different values of *n*_*i*_ when *d*_*i*_ = 70 nm by using (**b**) the TMM, and, (**d**) the FDTD method. Contour plots of the transmission spectra of the MIM filter (**e**) for varying values of *d*_*i*_ when *n*_*i*_ is 2.2, and, (**f**) for varying values of *n*_*i*_ when *d*_*i*_ is 70 nm, versus the wavelength, which are calculated by TMM, and, the output color as a function of wavelength is also shown at the bottom of the plots in (**e**) and (**f**). In all cases, the material of both metal layers is chosen as Ag and the layers are 25 nm thick.

To explore the effect of the thickness of the dielectric layer on the transmission spectrum, we refer to equation (1). The term *n*_*i*_*d*_*i*_ on the left-hand side of this equation is actually the optical beam path inside the cavity. The main approach to tune the transmission spectrum is to modulate the beam path. It is obvious from the equation that by increasing the path, the resonance wavelength or *λ*_*res*_ increases as well, or, in other words, it experiences a red-shift. Therefore, to tune the transmission spectrum, one can change either *d*_*i*_ or *n*_*i*_. Figure 5(a,c) show the transmission spectrum of the MIM structure for different thicknesses of the dielectric layer. The transmission profiles presented in Fig. 5(a) are calculated using the TMM method, and the ones shown in Fig. 5(c) are simulated by employing the Lumerical FDTD Solutions^48^. The metal layers are 25 nm thick and their material is chosen as Ag, and *n*_*i*_ is equal to 2.2. The red-shift in the transmission spectrum can be observed while increasing *d*_*i*_. Figure 5(b,d) demonstrate the transmission spectra of the structure for different values of *n*_*i*_, which are calculated using the TMM and the FDTD methods, respectively. *d*_*i*_ is assumed to be 70 nm thick and the metal layers are completely the same as the ones for Fig. 5(a). It can be seen that by increasing *n*_*i*_, the resonance wavelength of the transmission increases. To obtain a more precise image about the spectrum tuning effects, Fig. 5(e,f) depict the contour plots of the transmission spectrum versus the wavelength for varying values of *d*_*i*_ and *n*_*i*_, respectively. Both contour plots are calculated using the TMM method. The color corresponding to each wavelength is also shown at the bottom of Fig. 5(e,f) to help visualizing the change of the output color. It is seen from Fig. 5(f) that a less than twofold difference in *n*_*i*_ is required to cover the whole visible spectrum. However, the important restriction is that the above-mentioned tunings can be obtained when the changes are made at the stage of fabrication, i.e., by either changing the dielectric thickness or replacing the dielectric material with another dielectric with a different refractive index.

Here, by using an EO dielectric material, the refractive index of which changes while being under variations of applied voltage, the tunability of the transmission spectrum, like that in Fig. 5(b), can be realized in an active way after the fabrication. This is the basis of this work, which will be explored in the next section.

In order to design a color filter with a reasonably high quality factor (i.e., narrow bandwidth) and at the same time to have an appropriate transmission intensity, the choice of metals and the optimization of their thickness are of high importance. As it will be shown below, it is critical for the color filter performance.

**Figure 6:**
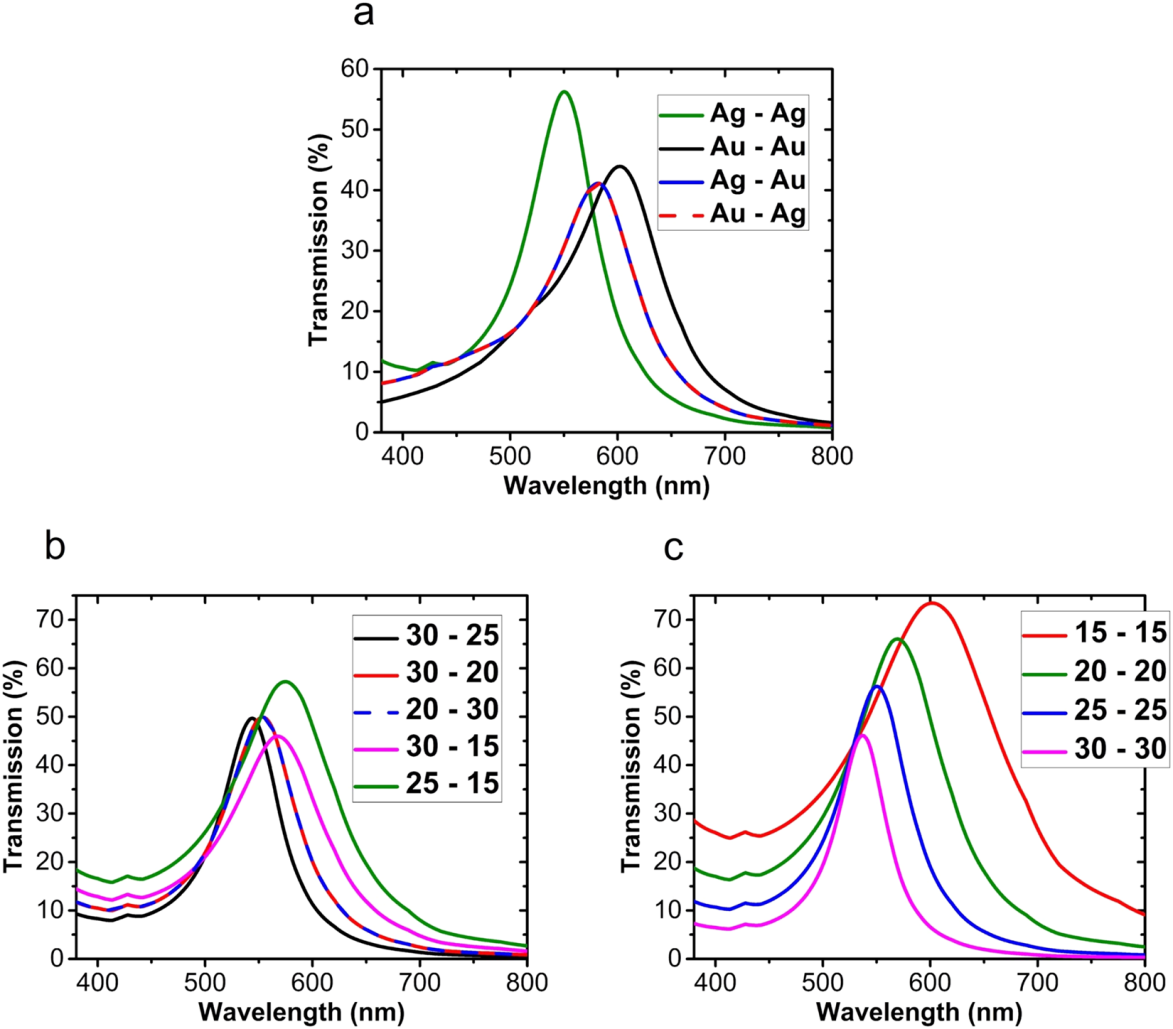
The transmission spectrum of the MIM filter calculated (**a**) for all possible combinations of Ag and Au used in the metal layers, and for different pairs of (**b**) unequal and (**c**) equal values of *d*_*t*_ and *d*_*b*_ when *d*_*i*_ is 70 nm and *n*_*i*_ is 2.2, and both the metal layers are chosen as 25 nm thick Ag.

Figure 6(a) depicts the transmission spectrum of the MIM structure for *n*_*i*_ and *d*_*i*_ being equal to 2.2 and 70 nm, respectively, in the case of using all possible four combinations of Silver (Ag) and Gold (Au) as the top and the bottom metal layers with the thickness of 25 nm. The two metal names in the legend of the figure, left and right ones, correspond to the top and the bottom metal layers, respectively. It can be seen that the best result is obtained in the case of using Ag for both the top and the bottom metal layers, both in terms of the transmission strength and the higher quality factor. It can be observed that by interchanging Au and Ag in the positions of the top and the bottom metal layers, the transmission spectrum remains the same. The complex refractive index data of Ag are taken from Palik^53^ (0–2 *µ*m) and for Au, they are taken from Johnson and Christy^54^.

Now, as the final step for the optimization of the structure, we investigate the effect of the thickness of the top and the bottom metal layers. Figure 6(b) shows the transmission spectrum for the case of ${d}_{t}\,\ne \,{d}_{b}$. The results also show that similarly to replacing one metal by another, like in Fig. 6(a), there is no difference when interchanging the thicknesses of the top and the bottom metal layers. Figure 6(c) demonstrates the calculated transmission of the structure when *d*_*t*_ = *d*_*b*_. It is obvious from Fig. 6(b,c) that by choosing thinner metal layers, the transmission amplitude increases, however, this comes at the cost of losing the quality factor and the desired  sharpness of the spectrum of the color filter. Taking this trade-off behavior into account, based on the calculations presented above, we choose the thickness of each of the top and the bottom metal layers to be 25 nm to achieve a reasonable compromise between the quality factor and transmission strength.

From Fig. 6 it is observed that by changing the thickness or the material of the metal layers, the resonance wavelength shifts as well. This may seem counter intuitive at first, but it can be clearly explained based on the previously presented results for the reflection phases, which are obtained from the circuit model. Indeed, any change in the choice of metal or the thickness of the top metal layer changes the values of *Z*_*t*_, *k*_*t*_, or *d*_*t*_. Subsequently, the value of *Z*_*in*,*t*_ (equation (2)) and therefore the values of *r*_*t*_ and especially *ɸ*_*t*_ (equations (3,4)) are changed. In a similar manner, any change in the bottom metal layer leads to a change in the value of *ɸ*_*t*_. Therefore, the changes of the metal layers modulate the values of reflection phase shifts, and this leads to that the resonance wavelength experiences a shift (see equation (1)).

To briefly summarize, we have so far optimized the color filter by choosing the appropriate metal to be Ag and the thickness of the top and bottom metal layers to be 25 nm, both. We also explained in-detail the effects exerted by the basic parameters on the quality factor, transmission intensity, and the resonance wavelength of this MIM filter.

## Electrical Tuning

As shown in the previous section, a less than twofold difference in *n*_*i*_ should be sufficient to cover the whole visible spectrum. The required difference can be realized by implementing the electrical tunability through the use of DAST, an EO material, as the dielectric layer. DAST has a large EO coefficient equal to 3.41 nm/V^43,55–57^. The refractive index of DAST is a function of the applied voltage and can be written as

5 $${n}_{i}={n}_{0}+\frac{dn}{du}(\frac{V}{{d}_{i}})$$

where *d*_*i*_ denotes the thickness of DAST layer sandwiched between the two metal layers and *V* is the applied voltage; $\frac{dn}{du}=3.41\,{\rm{n}}{\rm{m}}/{\mathtt{V}}$ is the EO coefficient with $u$ being the applied electric field, and *n*_0_ = 2.2 is the refractive index of DAST at *V* = 0.

**Figure 7:**
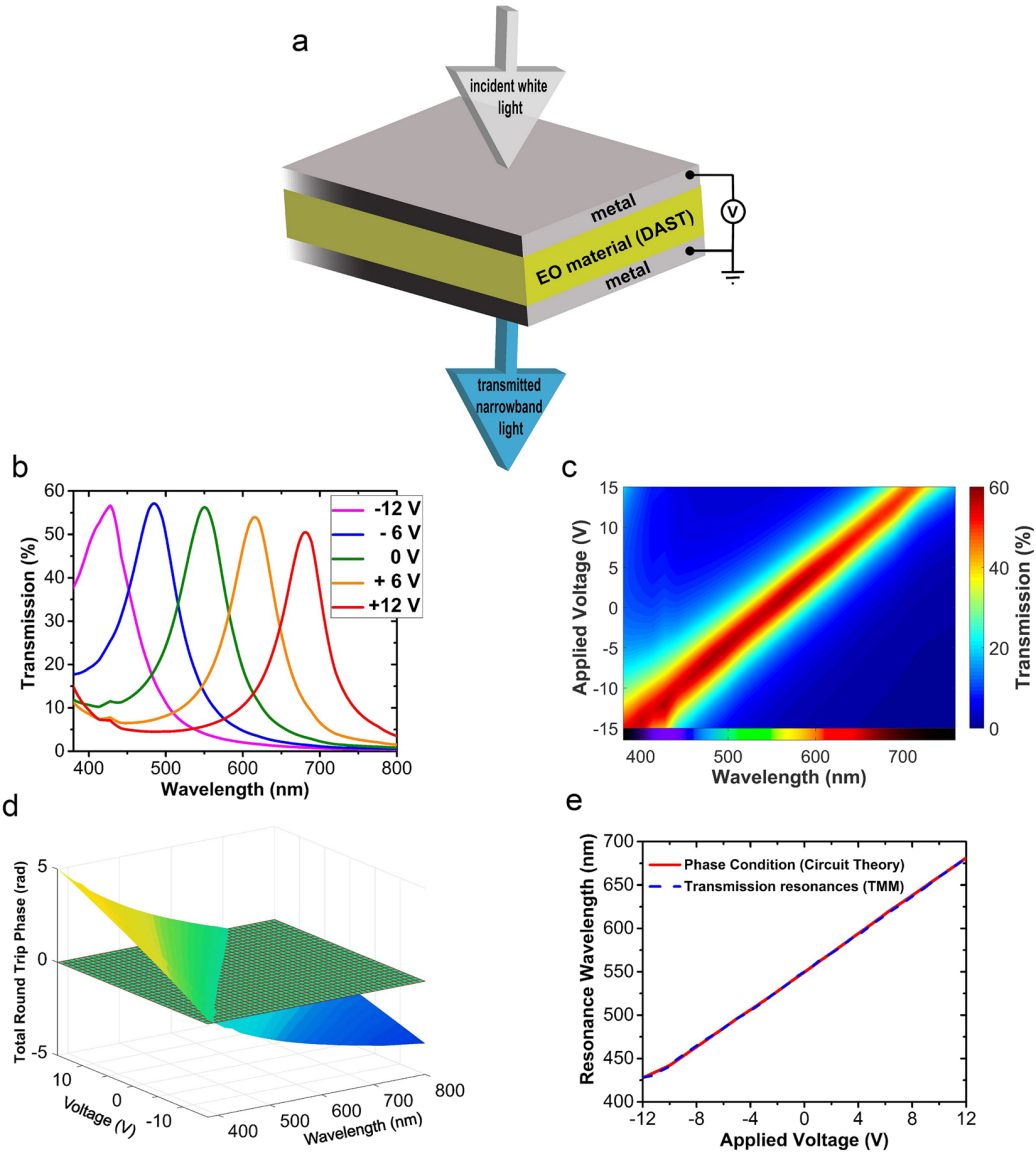
(**a**) 3D schematic of the electrically tunable color filter, (**b**) the transmission spectrum of the color filter for different values of the applied voltage, (**c**) contour plots of the transmission spectra at continuous variation of applied voltage, the output color as a function of wavelength is also shown at the bottom of the plot, (**d**) 3D surface plot of *ɸ*_*tot*_ versus wavelength and applied voltage; the meshed plane shows the zero-phase plane, and, (**e**) the resonance wavelengths versus the applied voltage calculated from the phase condition (equation 1) and from the TMM method.

Figure 7(a) depicts the schematic of the structure with applied voltage. Figure 7(b) shows the transmission spectrum for different values of the applied voltage when *d*_*i*_ is 70 nm. It is obvious from this figure that by varying the voltage from −12 to +12 volts almost the whole visible spectrum can be covered by the output transmission color of this MIM filter. Figure 7(c) demonstrates the continuous tuning of the transmission spectrum by plotting the contour plots of the transmission versus the wavelength and applied voltage. For a better visualization of the color tunability, the output color as a function of wavelength is also shown at the bottom of the plot of Fig. 7(c).

The 3D surface plot of the accumulated phase in a round-trip inside the cavity, *ɸ*_*tot*_, is shown in Fig. 7(d) as a function of wavelength and the applied voltage. The intersection of the surface corresponding to *ɸ*_*tot*_ with the plane of zero-phase (the first integer multiple of 2*π*) determines the points of resonance and, thus, the expected peaks of transmission spectra. It can be seen that the intersection with zero-phase plane (the resonance wavelength) increases from around 400 nm to around 700 nm by increasing the applied voltage. The resonance wavelength versus the voltage is plotted in Fig. 7(e). Here, the wavelengths are calculated by finding the peak of the transmission spectrum from the TMM method, and are also calculated from the intersection of *ɸ*_*tot*_ with zero-phase plane shown in Fig. 7(d). There is a perfect agreement between the two methods. The results presented in Fig. 7(e) clearly show that the fine and continuous tuning of the output color can be achieved by varying the applied voltage from −12 V to +12 V. Based on these results, the average shift in the resonance wavelength for every 10 V increment in applied voltage is 105.4 nm, which is a great number compared to the previous works^13,43,58,59^. For instance, in the design proposed in^59^, a less than 50 nm shift of the resonance wavelength is obtained in the visible regime by the application of 160 V. In the design proposed in^17^, a 40 nm shift is obtained with 50 V. Also in^46^, the resonance peak shifts 22.5 nm per 10 V. Finally, in^4^, a less than 100 nm shift is achieved in the near-infrared region by a 70 V applied voltage. Therefore, our proposed design is really power-efficient.

**Figure 8:**
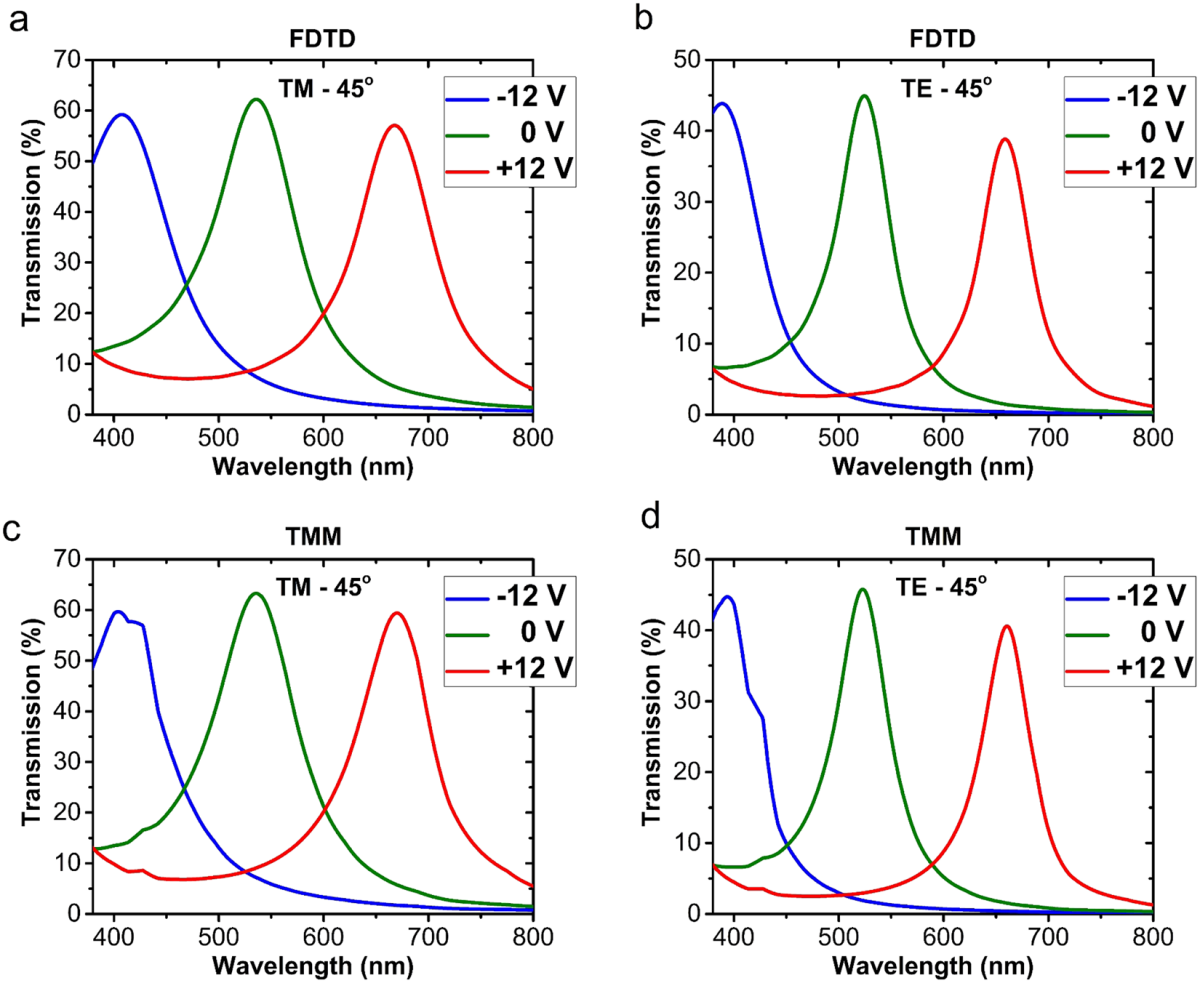
The transmission spectrum of the electrically tunable color filter for different values of the applied voltage at the incidence angle of 45 degrees, for (**a**) TM and (**b**) TE polarization, calculated by the FDTD method, and for (**c**) TM and (**d**) TE polarization, calculated by the TMM method.

It is also expected that the suggested structure has the potential of electrical tunability in the case of oblique incidence. By applying the changes that are mentioned in the Supplementary Information to the TMM calculations, the transmission spectrum of the structure for the incidence angle of 45 degrees has been calculated. The results are shown in Fig. 8 for both TM and TE polarizations. They confirm that the tunability is nearly the same as at normal incidence; i.e., almost all the visible range gets covered by sweeping the voltage between −12 and +12 volts.

The obtained results show that by the use of this simple geometry, and by the appropriate and optimized choice of materials and dimensions, a powerful tuning of the color of the MIM cavity based filter in the visible region can be obtained. The proposed structure covers the whole visible region by varying voltage only from −12 to +12 volts, so it is also power-efficient. This structure does not require any patterning and lithography and has a total thickness of 120 nm, i.e., it is ultra-thin and significantly light. Therefore, it is perspective for large-area and mass production of electrically tunable color filters.

## Conclusion

The general concept, theoretical background, and in-detail design steps of an MIM cavity based, electrically tunable color filter are presented. The effect of each design parameter has been investigated. An unprecedentedly simple approach has been suggested for the precise calculation of the resonance wavelength of the MIM structures, which is based on the circuit theory. The optimized electrically tunable structure which has the total thickness of 120 nm gets tuned from transmission output spectrum of the violet color to the red color, by only varying the applied voltage from −12 to +12 volts. The electrical tuning is continuous and covers the whole visible region. The proposed design has an important advantage of being lithography-free, which makes it a great candidate for large-area and mass production of tunable color filters. Our design is also relatively power-efficient compared to the earlier works. The presented structure can be used in a large number of applications such as optical communication and switching, displays, imaging devices, and color-tunable windows.

## Methods

FDTD Lumerical simulations^48^ are carried out in a 2D simulation environment in the (x, z)-plane (see Fig. 1(b)). The z-axis is normal to the structure and the x-axis is parallel to the interfaces of layers of the structure. Therefore, Perfectly Matched Layer (PML) boundary condition is used for the boundaries normal to the z-direction and periodic boundary condition is used for the boundaries normal to the x-direction. The simulation region or the unit cell has 200 nm size in the x-direction and 4000 nm size in the z-direction. The structure is centered inside the simulation region with respect to the mid-plane in z-direction. A plane wave source is placed above the structure, close to the top boundary of the simulation region, which is illuminating toward z-direction to the structure. A linear frequency-domain field and power monitor is placed on the other side of the structure, close to the bottom boundary of the simulation region to collect the transmitted light. The meshing applied to the structure has 2 nm steps in the z-direction and 5 nm steps in the x-direction.

## Electronic supplementary material

Supplementary Information

## References

[1] Xiang J (2015). Electrically Tunable Selective Reflection of Light from Ultraviolet to Visible and Infrared by Heliconical Cholesterics. Adv. Mat..

[2] Economou EC (2017). Electrically Tunable Open-Stub Bandpass Filters Based on NematicLiquid Crystals. Phys. Rev. Appl..

[3] Wang CT (2016). Full-color reflectance-tunable filter based on liquid crystal cladded guided-mode resonant grating. Opt. Express.

[4] Qian LY (2015). Optical notch filter with tunable bandwidth based on guided-mode resonant polarization-sensitive spectral feature. Opt. Express.

[5] Wang H (2009). Doped Organic Crystals with High Efficiency, Color-Tunable Emission toward Laser Application. Cryst. Growth Des..

[6] Pan F, McCallion K, Chiappetta M (1999). Waveguide fabrication and high-speed in-line intensity modulation in 4-N,N-4′-dimethylamino-4′-N′-methyl-stilbazolium tosylate. Appl. Phys. Lett..

[7] Thakur M, Xu JJ, Bhowmik A, Zhou LG (1999). Single-pass thin-film electro-optic modulator based on an organic molecular salt. Appl. Phys. Lett..

[8] Yao Y (2013). Broad Electrical Tuning of Graphene-Loaded Plasmonic Antennas. Nano Lett..

[9] AbdollahRamezani S, Arik K, Farajollahi S, Khavasi A, Kavehvash Z (2015). Beam manipulating by gate-tunable graphene-based metasurfaces. Opt. Lett..

[10] Tavakol MR, Saba A, Jafargholi A, Khavasi A (2017). Terahertz spectrum splitting by a graphene-covered array of rectangular grooves. Opt. Lett..

[11] Yao Y (2014). Electrically Tunable Metasurface Perfect Absorbers for Ultrathin Mid-Infrared Optical Modulators. Nano Lett..

[12] George D (2017). Electrically tunable diffraction efficiency from gratings in Al-doped ZnO. Appl. Phys. Lett..

[13] Mirshafieyan SS, Gregory DA (2018). Electrically tunable perfect light absorbers as color filters and modulators. Sci. Rep..

[14] Kim SJ, Brongersma ML (2017). Active flat optics using a guided mode resonance. Opt. Lett..

[15] Park J, Kang JH, Kim SJ, Liu XG, Brongersma ML (2017). Dynamic Reflection Phase and Polarization Control in Metasurfaces. Nano Lett..

[16] Yi F (2013). Voltage tuning of plasmonic absorbers by indium tin oxide. Appl. Phys. Lett..

[17] Park J, Kang JH, Liu XG, Brongersma ML (2015). Electrically Tunable Epsilon-Near-Zero (ENZ) Metafilm Absorbers. Sci. Rep..

[18] Huang YW (2016). Gate-Tunable Conducting Oxide Metasurfaces. Nano Lett..

[19] Forouzmand A, Mosallaei H (2016). Tunable two dimensional optical beam steering with reconfigurable indium tin oxide plasmonic reflectarray metasurface. J. Opt..

[20] Liang XY (2014). Colloidal Indium-Doped Zinc Oxide Nanocrystals with Tunable Work Function: Rational Synthesis and Optoelectronic Applications. Chem. Mat..

[21] Yamaguchi K, Fujii M, Okamoto T, Haraguchi M (2014). Electrically driven plasmon chip: Active plasmon filter. Appl. Phys. Express.

[22] Zheludev NI, Plum E (2016). Reconfigurable nanomechanical photonic metamaterials. Nat. Nanotech..

[23] Feigenbaum E, Diest K, Atwater HA (2010). Unity-Order Index Change in Transparent Conducting Oxides at Visible Frequencies. Nano Lett..

[24] Vukusic P, Sambles JR, Lawrence CR, Wootton RJ (2001). Structural colour - Now you see it now you don’t. Nature.

[25] Srinivasarao M (1999). Nano-optics in the biological world: Beetles, butterflies, birds, and moths. Chem. Rev..

[26] Vukusic P, Sambles JR (2003). Photonic structures in biology. Nature.

[27] Barrows FP, Bart MH (2014). Photonic Structures in Biology: A Possible Blueprint forNanotechnology. Nanomat. Nanotech..

[28] Huang JY, Wang XD, Wang ZL (2006). Controlled replication of butterfly wings for achieving tunable photonic properties. Nano Lett..

[29] Lee JH (2005). High ambient-contrast-ratio display using tandem reflective liquid crystal display and organic light-emitting device. Opt. Express.

[30] Belyaev VV (2015). Promising Applications and Technologies of Liquid Crystal Displays and Photonics Devices. Liq. Cryst. Appl..

[31] Koo HS, Chen M, Pan PC (2006). LCD-based color filter films fabricated by a pigment-based colorant photo resist inks and printing technology. Thin Solid Films.

[32] Li ZY, Butun S, Aydin K (2015). Large-Area, Lithography-Free Super Absorbers and Color Filters at Visible Frequencies Using Ultrathin Metallic Films. ACS Photonics.

[33] Gu YH, Zhang L, Yang JKW, Yeo SP, Qiu CW (2015). Color generation via subwavelength plasmonic nanostructures. Nanoscale.

[34] Park CS (2017). Structural Color Filters Enabled by a Dielectric Metasurface Incorporating Hydrogenated Amorphous Silicon Nanodisks. Sci. Rep..

[35] Zeng BB, Gao YK, Bartoli FJ (2013). Ultrathin Nanostructured Metals for Highly Transmissive Plasmonic Subtractive Color Filters. Sci. Rep..

[36] Kanamori Y, Shimono M, Hane K (2006). Fabrication of transmission color filters using silicon subwavelength gratings on quartz substrates. IEEE Photon. Technol. Lett..

[37] Lee KT, Seo S, Lee JY, Guo LJ (2014). Ultrathin metal-semiconductor-metal resonator for angle invariant visible band transmission filters. Appl. Phys. Lett..

[38] Proust J, Bedu F, Gallas B, Ozerov I, Bonod N (2016). All-Dielectric Colored Metasurfaces with Silicon Mie Resonators. ACS Nano.

[39] Vashistha V (2017). All-Dielectric Metasurfaces Based on Cross-Shaped Resonators for Color Pixels with Extended Gamut. ACS Photonics.

[40] Uddin MJ, Khaleque T, Magnusson R (2014). Guided-mode resonant polarization-controlled tunable color filters. Opt. Express.

[41] Ellenbogen T, Seo K, Crozier KB (2012). Chromatic Plasmonic Polarizers for Active Visible Color Filtering and Polarimetry. Nano Lett..

[42] Vashistha V, Vaidya G, Gruszecki P, Serebryannikov AE, Krawczyk M (2017). Polarization tunable all-dielectric color filters based on cross-shaped Si nanoantennas. Sci. Rep..

[43] Guo JJ (2017). Electrically Tunable Gap Surface Plasmon-based Metasurface for Visible Light. Sci. Rep..

[44] Park CH (2013). Electrically tunable color filter based on a polarization-tailored nano-photonic dichroic resonator featuring an asymmetric subwavelength grating. Opt. Express.

[45] Aalizadeh M, Khavasi A, Butun B, Ozbay E (2018). Large-Area, Cost-Effective, Ultra-Broadband Perfect Absorber Utilizing Manganese in Metal-Insulator-Metal Structure. Sci. Rep..

[46] Diest K, Dionne JA, Spain M, Atwater HA (2009). Tunable Color Filters Based on Metal-Insulator-Metal Resonators. Nano Lett..

[47] Lee BJ, Zhang ZM (2006). Design and fabrication of planar multilayer structures with coherent thermal emission characteristics. J. Appl. Phys..

[48] Lumerical Inc. http://www.lumerical.com/tcad-products/fdtd/.

[49] Staffaroni M, Conway J, Vedantam S, Tang J, Yablonovitch E (2012). Circuit analysis in metal-optics. Photon. Nanostruct. Fundam. Appl..

[50] Rodriguez-Ulibarri P, Beruete M, Serebryannikov AE (2017). One-way quasiplanar terahertz absorbers using nonstructured polar dielectric layers. Phys. Rev. B.

[51] Medina F, Mesa F, Skigin DC (2010). Extraordinary Transmission Through Arrays of Slits: A Circuit Theory Model. IEEE Trans. Microw. Theory Tech..

[52] Pozar, D. M. *Microwave engineering*. (Wiley, 2012).

[53] Palik, E. D. *Handbook of Optical Constants of Solids*. (Academic press, 1998).

[54] Johnson PB, Christy RW (1972). Optical Constants of the Noble Metals. Phys. Rev. B.

[55] Geis W (2004). Fabrication of crystalline organic waveguides with an exceptionally large electro-optic coefficient. Appl. Phys. Lett..

[56] Zhu, Y. J., Huang, X. G. & Mei, X. A Surface Plasmon Polariton Electro-Optic Switch Based on a Metal-Insulator-Metal Structure with a Strip Waveguide and Two Side-Coupled Cavities. *Chin*. *Phys*. *Lett*. **29** (2012).

[57] Taheri AN, Kaatuzian H (2015). Numerical investigation of a nano-scale electro-plasmonic switch based on metal-insulator-metal stub filter. Opt. Quant. Electron..

[58] Komar A (2017). Electrically tunable all-dielectric optical metasurfaces based on liquid crystals. Appl. Phys. Lett..

[59] Bibbo L (2017). Tunable narrowband antireflection optical filter with a metasurface. Photonics Res..

