# Supplementary material for: Toward Electrically Tunable, Lithography-Free, Ultra-Thin Color Filters Covering the Whole Visible Spectrum
Source: Sci Rep. 2018 Jul 27;8:11316. doi: 10.1038/s41598-018-29544-x (PMC6063855; doi:10.1038/s41598-018-29544-x)
Supplement: Supplementary file 1 — Supplementary Information [file 41598_2018_29544_MOESM1_ESM.pdf]

Toward Electrically Tunable, Lithography-Free, Ultra-Thin Color Filters Covering the Whole Visible Spectrum

Supplementary Information

Majid Aalizadeh[1,2*], Andriy E. Serebryannikov[3,4], Amin Khavasi[5], Guy A. E. Vandenbosch[3], and Ekmel Ozbay[1,2,6,7]

[1]Department of Electrical and Electronics Engineering, Bilkent University, Ankara 06800, Turkey
[2]Nanotechnology Research Center (NANOTAM), Bilkent University, Ankara 06800, Turkey
[3]ESAT-TELEMIC, Katholieke Universiteit Leuven, 3000 Leuven, Belgium
[4]Faculty of Physics, Adam Mickiewicz University, 61-614 Poznan, Poland
[5]Electrical Engineering Department, Sharif University of Technology, Tehran, 11155-4363, Iran
[6]National Nanotechnology Research Center (UNAM), Bilkent University, Ankara 06800, Turkey
[7]Department of Physics, Bilkent University, Ankara 06800, Turkey

***corresponding author: majid.aalizadeh@bilkent.edu.tr**

I) Transfer Matrix Method (TMM) calculations

In order to perform the TMM calculations, the electric and magnetic fields inside each layer of the structure are split into the forward (+z) and backward (-z) propagating waves, as follows:

$$E_i = E_i^+ e^{jk_i z} + E_i^- e^{-jk_i z}, \tag{S1a}$$

$$H_i = H_i^+ e^{jk_i z} + H_i^- e^{-jk_i z}, \tag{S1b}$$

where E_i^+ and H_i^+ denote the amplitude of the electric and magnetic fields, respectively, for the waves propagating forwardly, i.e., in the +z direction, and E_i^- and H_i^- denote the amplitudes for the backwardly propagating waves in the ith layer. The number indicating each layer, along with the names assigned to each interface (z_i) is shown in the schematic of the cross section of the structure in the (x,z)-plane, see Fig. S1. Finally, $k_i = k_0 n_i$ represents the propagation constant of the ith layer, $k_0 = \frac{\omega}{c}$ is the propagation constant of the free space, ω is the angular frequency, and c is the speed of light in the free space.

To calculate the transmission, one should evaluate the ratio of E_4^+ to E_0^+. The amplitudes of the fields in each layer are related to each other by imposing boundary conditions. The matrix that determines the relation between the fields at the interface of ith and $(i+1)^{\text{th}}$ layer is named $\mathrm{I}_{i,i+1}$. To obtain this matrix, we start with the boundary conditions at the ith interface (see Fig. S1(b)). As long as the structure is large-area, it does not have polarization dependence for normal incidence. Therefore, both the electric and the magnetic field are tangential to the interface at the normal incidence. The tangential components of the fields must be continuous at the source-free interfaces. Therefore, we can write the boundary conditions as follows:

$$(E_i^+ + E_i^-)\Big|_{z=z_i} = (E_{i+1}^+ + E_{i+1}^-)\Big|_{z=z_i}, \tag{S2a}$$

$$(H_i^+ + H_i^-)\Big|_{z=z_i} = (H_{i+1}^+ + H_{i+1}^-)\Big|_{z=z_i} . \tag{S2b}$$

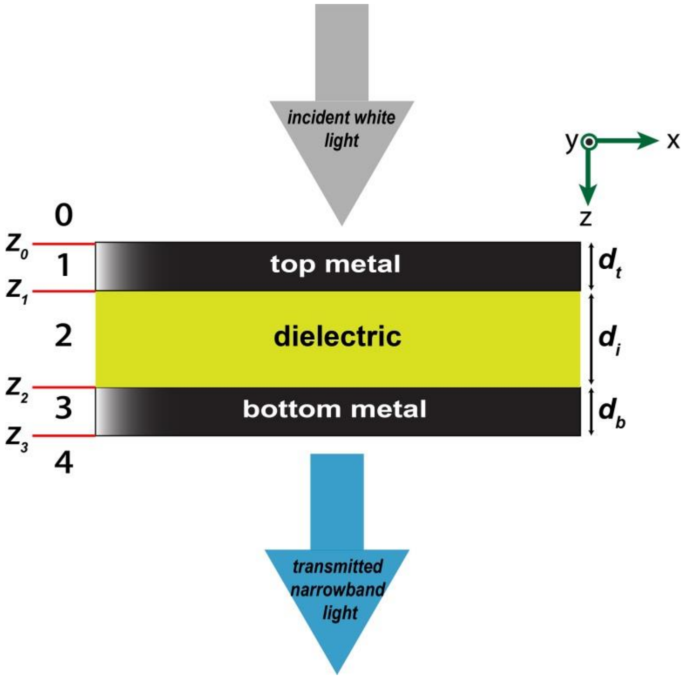

Figure S1. Cross section of the structure in the (x,z)-plane

Then, by substitution of the magnetic field with the electric field from the equation of $H = \frac{n}{Z_0} E$, in which Z_0 is the impedance of the free space, equation (S2b) can be written as follows:

$$(n_i\, E_i^+ - n_i\, E_i^-)\Big|_{z=z_i} = (n_{i+1}\, E_{i+1}^+ - n_{i+1}\, E_{i+1}^-)\Big|_{z=z_i} . \tag{S3}$$

From equations (S2a) and (S3), the interface matrix which relates electric-field amplitudes of the forwardly and backwardly propagating waves at two sides of the ith interface and is named $I_{i,i+1}$ can be obtained as follows:

$$\begin{pmatrix} E_{i+1}^+ \\ E_{i+1}^- \end{pmatrix}\Bigg|_{z=z_i} = \begin{pmatrix} \dfrac{n_{i+1}+n_i}{2n_{i+1}} & \dfrac{n_{i+1}-n_i}{2n_{i+1}} \\ \dfrac{n_{i+1}-n_i}{2n_{i+1}} & \dfrac{n_{i+1}+n_i}{2n_{i+1}} \end{pmatrix} \begin{pmatrix} E_i^+ \\ E_i^- \end{pmatrix}\Bigg|_{z=z_i} . \tag{S4}$$

Now, the matrix that stands for the propagation of the fields inside each layer, which is named as P_i, can be written as

$$\begin{pmatrix} E^{+}_{(z_i)} \\ E^{-}_{(z_i)} \end{pmatrix} = \begin{pmatrix} e^{j\beta_i d_i} & 0 \\ 0 & e^{-j\beta_i d_i} \end{pmatrix} \begin{pmatrix} E^{+}_{(z_{i-1})} \\ E^{-}_{(z_{i-1})} \end{pmatrix}. \tag{S5}$$

Next, the relation between the fields inside the first and the last layer can be written by sequentially multiplying the interface and propagation matrices as follows:

$$\begin{pmatrix} E_4^{+} \\ E_4^{-} \end{pmatrix} = \left(\mathrm{I}_{3,4}\right)\left(\mathrm{P}_3\right)\left(\mathrm{I}_{2,3}\right)\left(\mathrm{P}_2\right)\left(\mathrm{I}_{1,2}\right)\left(\mathrm{P}_1\right)\left(\mathrm{I}_{0,1}\right) \begin{pmatrix} E_0^{+} \\ E_0^{-} \end{pmatrix}, \tag{S6}$$

$$\begin{pmatrix} E_4^{+} \\ E_4^{-} \end{pmatrix} = \mathrm{M}_{2\times 2} \begin{pmatrix} E_0^{+} \\ E_0^{-} \end{pmatrix}. \tag{S7}$$

As shown in equation (S7), the matrix relating the fields at the first and the last interface, which is named as M, is equal to the multiple of the interface and propagation matrices; see equation (S6).

Finally, the transmission (T) can be calculated from the equation

$$\mathrm{T} = \left| \frac{E_4^{+}}{E_0^{+}} \right|^2 . \tag{S8}$$

Now, since only the ratio of the amplitudes is important for calculation of the transmission, the value of the amplitude of the incident wave, E_0^{+}, is assumed to be 1. It is also known that the value of E_4^{-} is zero, because there is no reflected wave after the last layer of the structure. Therefore, equation (S7) evolves into

$$\begin{pmatrix} E_4^{+} \\ 0 \end{pmatrix} = \begin{pmatrix} m_{11} & m_{12} \\ m_{21} & m_{22} \end{pmatrix} \begin{pmatrix} 1 \\ E_0^{-} \end{pmatrix}. \tag{S9}$$

Equation (S9) is equivalent to two linear equations with two unknowns, E_4^{+} and E_0^{-}. Therefore, by combining the equations (S8) and (S9), the transmission can be written as follows:

$$\mathrm{T} = \left| \frac{m_{11}m_{22} - m_{12}m_{21}}{m_{22}} \right|^2 . \tag{S10}$$

In order to calculate the transmission spectrum using the TMM method for the oblique incidence, the parameter k_i in the propagation matrix, P_i (equation (S5)), should be replaced by $k_i \cos\theta_i$, where θ_i is the angle between the direction of propagation and the plane normal to the interfaces, inside the ith layer. One more change to be done is to re-write the interface matrix for TE and TM polarizations as follows:

$$\mathrm{I}_{i,i+1}\Big|_{TE} = \begin{pmatrix} \dfrac{n_{i+1}\cos\theta_{i+1} + n_i\cos\theta_i}{2n_{i+1}} & \dfrac{n_{i+1}\cos\theta_{i+1} - n_i\cos\theta_i}{2n_{i+1}} \\ \dfrac{n_{i+1}\cos\theta_{i+1} - n_i\cos\theta_i}{2n_{i+1}} & \dfrac{n_{i+1}\cos\theta_{i+1} + n_i\cos\theta_i}{2n_{i+1}} \end{pmatrix}, \tag{S11a}$$

$$\mathrm{I}_{i,i+1}\Big|_{TM} = \begin{pmatrix} \dfrac{n_i\cos\theta_{i+1} + n_{i+1}\cos\theta_i}{2n_i} & \dfrac{n_i\cos\theta_{i+1} - n_{i+1}\cos\theta_i}{2n_i} \\ \dfrac{n_i\cos\theta_{i+1} - n_{i+1}\cos\theta_i}{2n_i} & \dfrac{n_i\cos\theta_{i+1} + n_{i+1}\cos\theta_i}{2n_i} \end{pmatrix}. \tag{S11b}$$

II) Optical and transmission properties of Silver and Gold

Since the proposed structure works in the transmission mode, looking at the optical properties and also the transmission profile of single layers of Ag and Au, which has been used in the material optimization procedure in the main paper, can give some insight about the transmission properties of single layers of these metals. The thickness of single layers for calculation of transmission has been taken as 25 nm which is the optimized thickness of both the top and bottom metal layers of the MIM filter. Figs. S2(a) and (b) show the refractive index and the light transmission from a 25 nm layer of these metals, respectively.

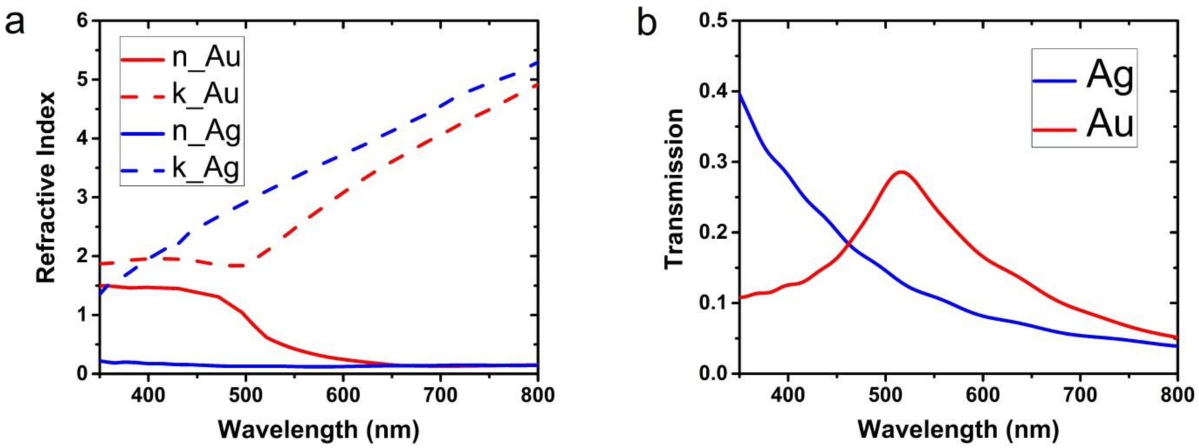

Figure S2. (a) Refractive index of Ag (blue) and Au (red). Real and imaginary parts are shown by solid and dashed lines, respectively. (b) Transmission calculated from a 25 nm single layer of Ag (blue) an Au (red)
